# A Simulation Analysis of Maternal Pelvic Floor Muscle

**DOI:** 10.3390/ijerph182010821

**Published:** 2021-10-15

**Authors:** Rongrong Xuan, Mingshuwen Yang, Yajie Gao, Shuaijun Ren, Jialin Li, Zhenglun Yang, Yang Song, Xu-Hao Huang, Ee-Chon Teo, Jue Zhu, Yaodong Gu

**Affiliations:** 1 The Affiliated Hospital of Medical School of Ningbo University, Ningbo 315020, China; fyxuanrongrong@nbu.edu.cn; 2 Key Laboratory of Impact and Safety Engineering (Ningbo University), Ministry of Education, Ningbo 315010, China; 1711042015@nbu.edu.cn (M.Y.); xuhao_huang@sjtu.edu.cn (X.-H.H.); 3 School of Medicine, Ningbo University, Ningbo 315211, China; 1811101128@nbu.edu.cn (Y.G.); 1911105135@nbu.edu.cn (S.R.); 2011105153@nbu.edu.cn (J.L.); 2011105155@nbu.edu.cn (Z.Y.); 4 Doctoral School on Safety and Security Sciences, Obuda University, 1034 Budapest, Hungary; yang.song@uni-obuda.hu; 5 School of Mechanical and Aerospace Engineering, Nanyang Technological University, Singapore 637459, Singapore; mecteo@ntu.edu.sg; 6 Faculty of Sports Science, Ningbo University, Ningbo 315211, China

**Keywords:** pelvic floor disorders, MRI, finite element analysis, delivery

## Abstract

Pelvic floor disorder (PFD) is a common disease affecting the quality of life of middle-aged and elderly women. Pelvic floor muscle (PFM) damage is related to delivery mode, fetal size, and parity. Spontaneous vaginal delivery causes especially great damage to PFM. The purpose of this study was to summarize the characteristics of PFM action during the second stage of labor by collecting female pelvic MRI (magnetic resonance imaging) data and, further, to try to investigate the potential pathogenetic mechanism of PFD. A three-dimensional model was established to study the influence factors and characteristics of PFM strength. In the second stage of labor, the mechanical responses, possible damage, and the key parts of postpartum lesions of PFM due to the different fetal biparietal diameter (BPD) sizes were analyzed by finite element simulations. The research results showed that the peak stress and strain of PFM appeared at one-half of the delivery period and at the attachment point of the pubococcygeus to the skeleton. In addition, during the simulation process, the pubococcygeus was stretched by about 1.2 times and the levator ani muscle was stretched by more than two-fold. There was also greater stress and strain in the middle area of the levator ani muscle and pubococcygeus. According to the statistics, either being too young or in old maternal age will increase the probability of postpartum PFM injury. During delivery, the entire PFM underwent the huge deformation, in which the levator ani muscle and the pubococcygeus were seriously stretched and the attachment point between the pubococcygeus and the skeleton were the places with the highest probability of postpartum lesions.

## 1. Introduction

Pelvic floor disorders (PFDs) have plagued most middle-aged and elderly women in the world. The specific symptoms of PFDs include pelvic organ prolapse (POP), stress urinary incontinence (SUI), myofascial syndrome, pelvic pain, ect. In the study of Delancey et al. [1], multiplanar proton density magnetic resonance images (MRI) were obtained from 80 nulliparous and 160 vaginally primiparous women by magnetic resonance (MR) at 0.5 cm intervals. From these images, no levator ani defects were identified in nulliparous women. However, among vaginally primiparous women, 32 women (20%) suffered from a visible defect in one or both levator ani muscles and, of these, 29 women (18%) were affected in the pubovisceral portion of the muscle and 3 women (2%) were affected in the iliococcygeal portion. Of the 32 women with defects, 23 women (71%) had SUI.

The causes of PFD can be divided into intrinsic and extrinsic factors [2]. Intrinsic factors include abnormal collagen matrix foundation, genetics, and ethnicity, etc. Chen et al. [3] found that the collagen content of pubic cervical fascia in women with SUI was less than that healthy females. The study of Jack et al. [4] showed that the incidence of organ prolapse was significantly higher in those women from the families in which organ prolapse have occurred in the members. Graham et al. [5] found that pelvic floor strength was different in women of different races. For example, Asian women had thicker pelvic floor ligaments and fascia than Caucasian women. Extrinsic factors include childbirth, hysterectomy, occupation, hormone replacement therapy, co-morbidities, etc. Childbirth was considered as the major factor. According to the study of Sze et al. [6], prolapse evaluation was conducted at 36th antepartum week and 6th postpartum week in women who had spontaneous vaginal delivery or caesarean section. The results showed that the caesarean group had a pelvic prolapse rate of approximately 3%, and the incidence of natural birth was up to 9%. In addition, hysterectomy would damage the endopelvic fascia, the support of the uterosacral-cardinal ligament, and the local nerve supply, thereby injuring the pelvic floor and triggering organ prolapse. Woodman et al. [7] found that among the people who suffered from the organ prolapse surgery, factory workers accounted for the largest proportion, followed by housewives, service workers, technical workers, and professionals. This shows that high-intensity labor also will cause pelvic floor disease.

Maternal labor is divided into three stages medically, namely the first, the second, and the third stages. The first stage of labor is the process from the beginning of regular uterine contractions to the end with complete cervical dilatation at 10 cm, and also known as “cervical dilation period”; this process usually takes more than 10 h. The second stage of labor begins with complete cervical dilatation and ends with the delivery of the fetus, and also known as “fetal delivery period”. The third stage of labor is defined by the period between the delivery of the fetus and the delivery of the placenta and fetal membranes, also known as “placenta delivery period”, this process usually takes 5 to 10 min. Clinical trials have shown that proper stretching of perineal tissue before and during childbirth can reduce the probability of perineal trauma. According to the study of Beckmann et al. [8], the flexibility of perineal tissues of women who had received perineal massage was significantly increased compared with that of women who hadn’t receive massage in the late pregnancy. During the second stage of labor, hyaluronidase (HAase) is injected into the perineal area to prevent maternal trauma. About 3 to 4 min after HAase injection, the perineal tissue will be softer and become more flexible, which will help the fetus to pass through the vaginal [9].

Female pelvic floor muscles (PFM) have multiple layers of complex anatomical structures, including several closely arranged muscles. Undoubtedly, it is very difficult to conduct the 3D modeling directly by the modeling software directly, so it is necessary to find new auxiliary means. Janda et al. [10] obtained a 3D geometrical data of pelvic cavity including muscle fibre directions by using a palpator device. They confirmed muscle fibre length, weight, volume, mean fiber length, mean sarcomere length, number of sarcomere, optimum fibre length, and other muscle data, and established a 3D mathematical modeling model based on experimental data of pelvic layer. For the model with non-uniform rational B-splines (NURBs) surfaces, the scanning method is generally adopted to extract the model. The currently known methods include magnetic resonance imaging (MRI), diffusion tensor imaging (DTI), computed tomography (CT), ultrasound, and so on. Among them, MRI has the advantage of avoiding ionizing radiation and has proven to be valuable in the characteristic manifestations of PFM defects [1,11]. DTI can also provide new help for the study of pelvic floor diseases. Several scientists [12,13,14] have demonstrated the feasibility of DTI in the modeling of normal female pelvic muscles. For example, Zijtaet al. [14] collected DTI data of five young female nulliparous subjects, and calculated the eigenvalues, fractional anisotropy, and average diffusivity from the fibre trajectories. The imaging resulted in a satisfactory anatomical representation of the pubovisceral muscle, perineal body, anal and urethral sphincter complex, and internal obturator muscle.

The constitutive relation is used to describe the mechanical behavior of a material. In the second stage of labor, women’s PFM undergo significant deformation. For materials undergoing large deformation, super-elastic constitutive models can be used, such as neo-Hookean constitutive relation, Mooney-Rivlin constitutive relation, and exponential constitutive relation. In the study of Brandao et al. [15], pelvic organs were described as hyperelastic materials, and the skeleton, the main supporting structure, was defined as a rigid body. In the second stage of simulation, muscle damage and structural damage was simulated by reducing the hardness of the material. Berardi [16] used the anisotropic hyperelastic constitutive model to describe PFM, assuming that the collagen fibers could not withstand compression load, since the fibers would bend under compression and the strain of the fibers was specified as positive, and the anisotropic component of the strain energy function came into play. Jing et al. [17] adopted the anisotropic viscoelastic neo-Hookean constitutive relation to obtain the convergent solution of six parameters of the material through biaxial test experiment and nonlinear regression algorithm. Martins et al. [18] believed that skeletal muscle has complex mechanical behaviors, which are basically incompressible, transversely isotropic, and highly non-linear, and thus established a passive hyperelastic model. While up to now, constitutive relation of PFM, which is used into the ordinary commercial software for numerical simulation, has also not been reported, in simulations the linear elastic relationship of stress and strain is adopted.

According to the investigation [19], it can be found that delivery causes great damage to the maternals’ PFM. The biomechanical analysis of the delivery process is helpful to study the mechanism of PFM injury. Therefore, our research intends to study the characteristics of PFM action by collecting female pelvic MRI data. A three-dimensional model is established to study the influence factors and damage mechanism of PFM, numerically analyze the stress and strain of PFM in the second stage of labor, and evaluate the damage on PFM and the key parts of postpartum PFM lesions due to the different fetal biparietal diameter (BPD) size, to provide empirical support for the medical community to prevent pelvic floor lesions, and to determine proper treatment methods.

## 2. Methods

### 2.1. Model Construction

This study has been approved by the Ethics Committee of School of Medicine of Ningbo University. After receiving the informed consent from the participant, the MRI Cor CUBE T2 scanning method was used to obtain her PFM images and DICOM data files at 0.5 mm scanning intervals. The DICOM data files are imported into the 3D Slicer case database, and then load the images into the horizontal slice view, sagittal slice view, and coronal slice view [20]. It can be found that the MRI scan area includes the entire pelvic cavity. In addition to PFM, there are other muscles, organs, and bones in the model [15,21,22]. Ignoring other organs and bone [23], only the mechanical response of PFM during the second stage of labor is concerned. A professional MRI physician who has more than ten years’ work experience helped to outline PFM from every slicer. The three-dimensional model of PFM was established in software 3D Slicer according to the following steps.

At first, a new segment is added in the segmentations module, and then edited in the segment editor module. Before editing the segment, it is important to properly select the master volume model, for multiple sets of MRI data would be imported and edited based on the original master volume model to generate new mapping volume data. On the contrary, the desired model cannot be obtained. Then we used the editing tools to extract the volume model of PFM. The brush tool (Paint) was used to select data. Since the thickness of PFM is very tiny, and the boundaries in the image are not obvious, it should be manually selected layer by layer. Under the guidance of a professional MRI physician, PFM is determined and selected layer by layer on the horizontal section window by a ball brush with a diameter of 1–2 mm. After selection of PFM, there are many gaps inside the model and many fragments around the model are found. Obviously, the model needs to be post-processed. The closing function in the smoothing module was set to fill the gaps in the model automatically. The opening module was set to remove external fragments. Furthermore, the joint smoothing function can make the model surface smoother.

**Figure 1:**
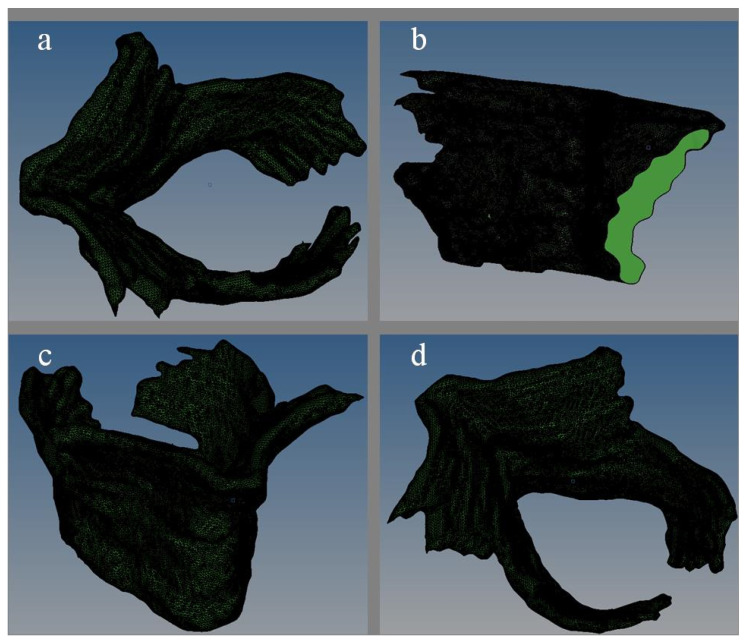
The three-dimensional solid diagram of pelvic floor muscle ((**a**) plan view; (**b**) side view; (**c**) lateral elevation; (**d**) side plan view).

Usually, the models obtained by scanning only consist of lines or elements. The software 3D Slicer can only output files in STL format (that is, the models are all made of triangular elements, not entities). So, the model must be materialized. The software Hypermesh is used to reversely conduct the solid modeling. In the software, the surface was formed by the elements, and then the 3D solid model is generated from the surface. Before being imported into Hypermesh, the STL files format should be converted into STP file format by ProE. In Hypermesh, all of the element units in the geometry module are selected to create the 2D mesh (from FE). In this process, it is necessary to notice that surfaces or entity elements, such as the tetrahedral element and hexahedral element, are not allowed to establish surface or solid except for 2D element. Then, surfaces are selected and bounding surfaces are generated in the geometric module. It should be emphasized that the selected surface must be closed and not have any open parts. The finally obtained solid model is shown in Figure 1.

### 2.2. Load and Boundary Conditions

Abaqus is employed to simulate the second stage of labor. In this paper, a sphere is established to simulate the fetal head [16,24]. Considering that the Young’s modulus and density of bone is much larger than that of muscle, the fetal head model is thus approximately defined as rigid body. The purpose of this study is to explore the influence of fetal biparietal diameter on PFM damage in the second stage of labor [17]. According to the investigation data, three spherical diameters of fetal head are chosen as in Table 1. The dimensions of PFM model are also shown in Table 1.

Isotropic linear elastic constitutive model was used in this paper. The material parameter of pelvic muscle from Hoyte [24] was adopted. And the material parameters of fetal skull were referred to the experimental results of Fung [25]. It is known that the Asian newborn’s weight is about 3 kg and the normal biparietal diameter is about 90 mm. Assuming that the fetal weight is all concentrated in the head, the approximate density can be calculated according to the density calculation formula. Specific material data is shown in Table 2.

**Figure 2:**
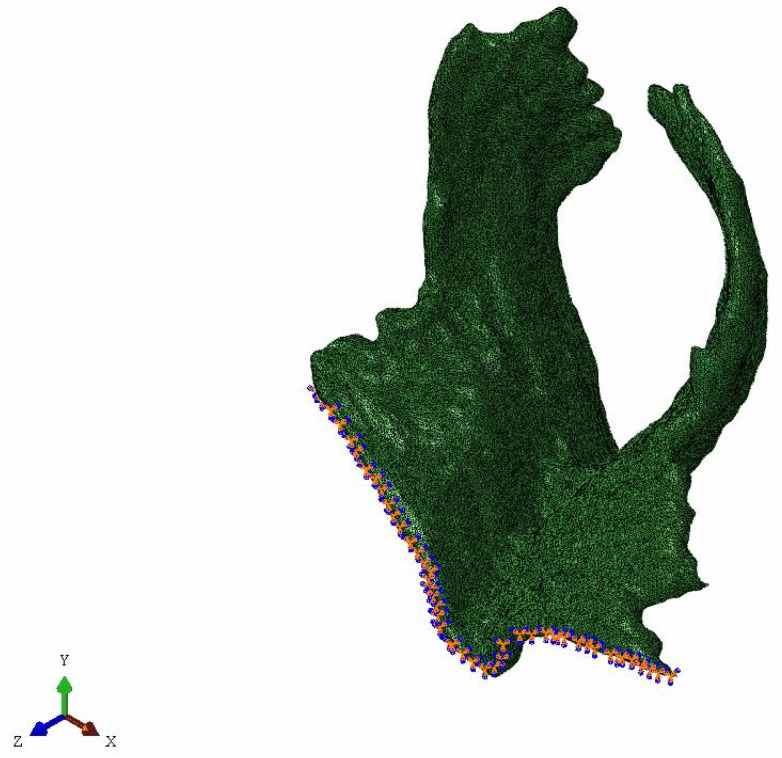
The constrained pelvic floor muscle.

This research is based on biomedical finite element simulation, so specific constraints of the model need to be combined with anatomical or clinical knowledge. Physiologically, muscles are attached to bones, so the binding point is usually at the joint connecting muscle or tendon with bone. However, it is difficult to determine the binding point of PFM, since they are actually a group of muscles which are composed of many complex muscles and connective tissue in a criss-cross formation. In addition, other pelvic organs, such as the bladder and rectum, may also make influence on PFM. In this paper, the upper margin of PFM was constrained, which was that freedom degrees of the outer margin of pubococcygeus muscles are all constrained [15,16,17,26]. The specific constraints are shown in Figure 2.

**Figure 3:**
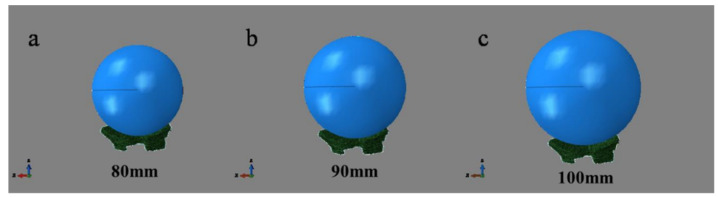
Assembly diagram of fetal head with different biparietal diameters and pelvic floor muscle.

In the initial condition setting, the fetal skull is set as the vertically down move under its gravity. Universal contact is adopted due to the complex structure of this model, and the surface of PFM is not smooth [15]. In the simulation of Berardi et al. [16], a friction coefficient of 0.03 was set. Friction coefficient of 0.03 was defined in this simulation too. Due to the uneven surface of PFM model, the fetal head model does not fit on the muscle surface perfectly. Hence, in order to avoid the interference of subsequent calculation units, the fetus head should be assembled as close to PFM surface as possible. Figure 3 is the assembly diagram of the fetus head with biparietal diameters of 80 mm, 90 mm, and 100 mm.

**Figure 4:**
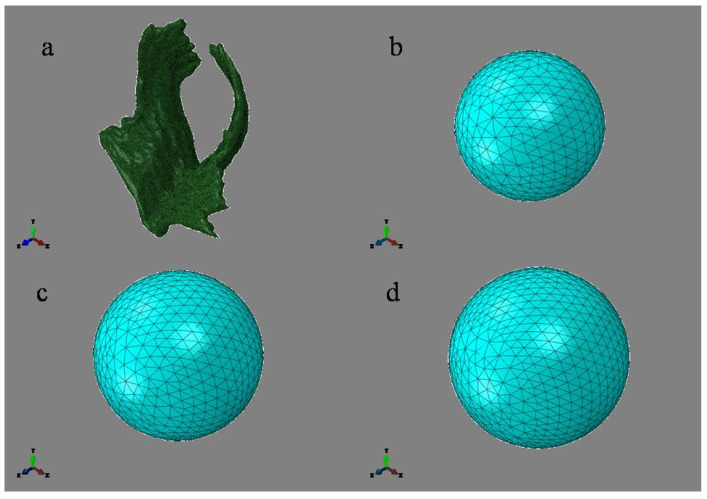
The part diagram with element division ((**a**) Pelvic floor muscle, 114,216 nodes, 515,633 elements; (**b**) 80 mm, 10,367 nodes, 6901 elements; (**c**) 90 mm, 15,822 nodes, 10,702 elements; (**d**) 100 mm, 19,703 nodes, 13,359 elements).

The simulation of the movements of the fetus during delivery started from where the fetal head is 23 mm away from the levator ani muscle (defined as the initial position of the fetal head). After the internal rotation, the simulation ended when the fetal head completed an extension of 90°. This study simulated the process of the fetal vertical downward movement under the action of gravity. The meshing of the muscle model was completed in Hypermesh. All components were divided by tetrahedral meshes, as shown in Figure 4. The surface and edge of PFM were smoothed, and finally the hexahedral elements were used to divide the element [27]. By comparing three meshes of different sizes (1.5 mm, 2 mm and 3 mm), a mesh of 2 mm was finally adopted [16,26,27].

## 3. Results

**Figure 5:**
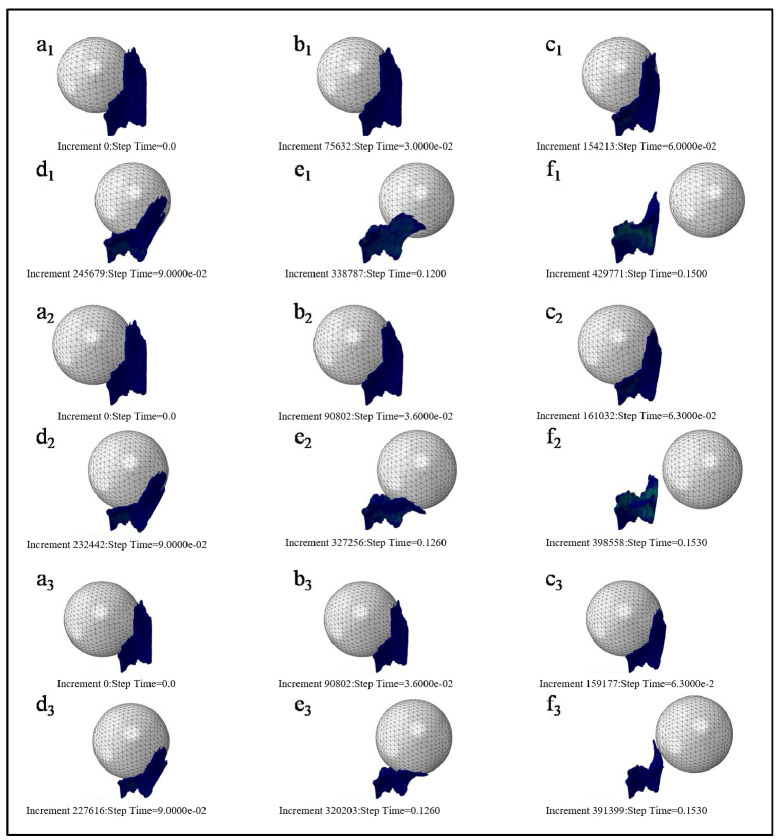
Motion status of three kinds of fetal head models at the different simulation time. ((**a**)--(**f**） refer to the motion status of 3 kinds of fetal head models (Models D80, D90, and D100 in order) at simulation time of 0 s, 0.03 s, 0.06 s, 0.09 s, 0.12 s, and 0.15 s).

Figure 5 describes the motion state at six different time points in the whole simulation process. The left end edge of PFM is fixed, and the motion status of 3 kinds of fetal head models is at simulation time of 0 s, 0.03 s, 0.06 s, 0.09 s, 0.12 s, and 0.15 s, respectively. Figure 5(a1–f1), Figure 5(a2–f2) and Figure 5(a3–f3) are, respectively, the motion states of Model D80, Model D90, and Model D100.

**Figure 6:**
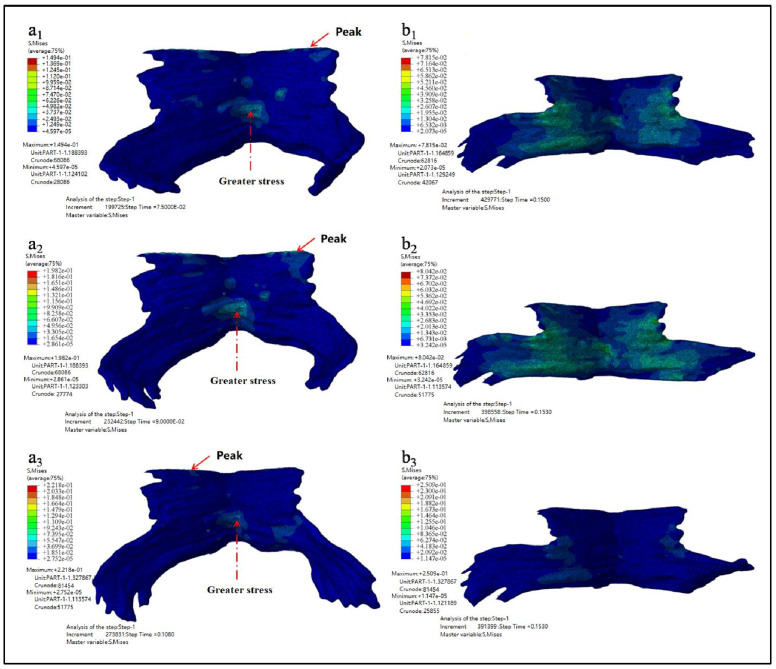
Equivalent stress nephogram (**a1**–**a3** show the equivalent stress nephogram of Models D80, D90, and D100 while **b1**–**b3** show the equivalent stress nephogram of Models D80, D90, and D100 at simulation time of 0.15 s).

In order to clearly observe the deformation of PFM, the fetal head model was hidden during post-treatment. Figure 6 shows the maximum equivalent stress nephogram of PFM and the equivalent stress nephogram at the end of simulation. Figure 6(a1–a3) show the equivalent stress nephogram of Models D80, D90, and D100. It is found that the stress peak appears at the point of the straight-line arrow, while the stress value appears at the point of the dotted line arrow; Figure 6(b1–b3) show the equivalent stress nephogram of Models D80, D90, and D100 at simulation time of 0.15 s.

The maximum equivalent stress, the occurrence time of the maximum equivalent stress, and the equivalent stress value at the end of simulation are shown in Table 3.

**Figure 7:**
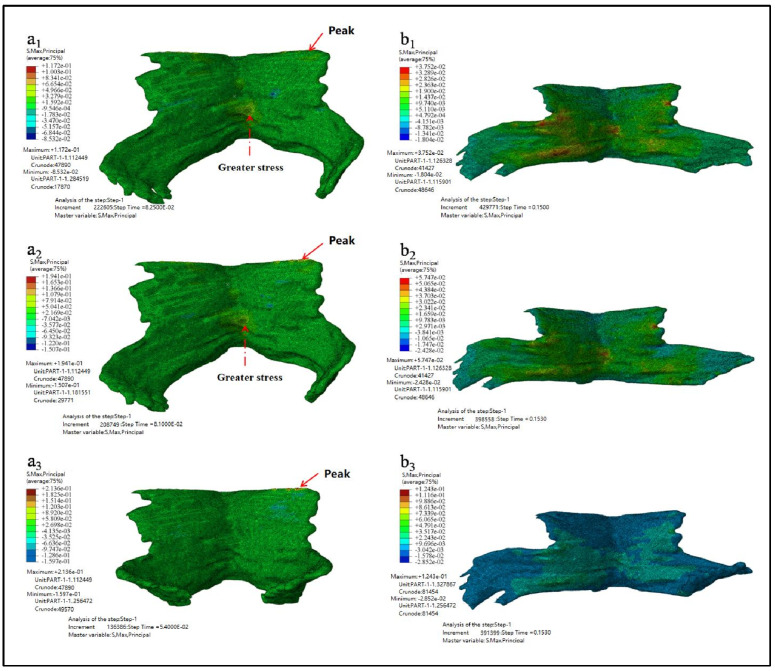
The maximum principal stress nephogram. (**a1**–**a3** showed the maximum principal stress nephogram of Models D80, D90 and D100 while **b1**–**b3** showed the principal stress nephogram of Models D80, D90, and D100 at the simulation time of 0.15 s).

Figure 7 showed the maximum principal stress nephogram of PFM and the principal stress nephogram at the end of simulation. Figure 7(a1–a3) showed the maximum principal stress nephogram of Models D80, D90 and D100. It showed that the stress peak appears at the point of the straight-line arrow, while the stress value appears at the point O the dotted line arrow. Figure 7(b1–b3) showed the principal stress nephogram of Models D80, D90, and D100 at the simulation time of 0.15 s.

The maximum principal stress, the occurrence time of the maximum principal stress, and the principal stress value at the end of simulation are shown in Table 4.

As can be seen from the results listed in Table 3 and Table 4 above, the larger the biparietal diameter of the fetus is, the larger the maximum equivalent stress and the maximum principal stress of PFM are. By observing the equivalent stress nephogram and the principal stress nephogram, and combining with the knowledge of the anatomy of PFM, we found that the peak value occurred at the joint point of the pubococcygeus muscle to the skeleton (the straight-line arrow), followed by the middle and lower part of the middle fissure of the iliac tail seam (the dotted line arrow). At the end of the simulation, that is, when *t* = 0.15 s, the intermediate region between the levator ani and the pubococcygeus muscles is subjected to larger stress.

**Figure 8:**
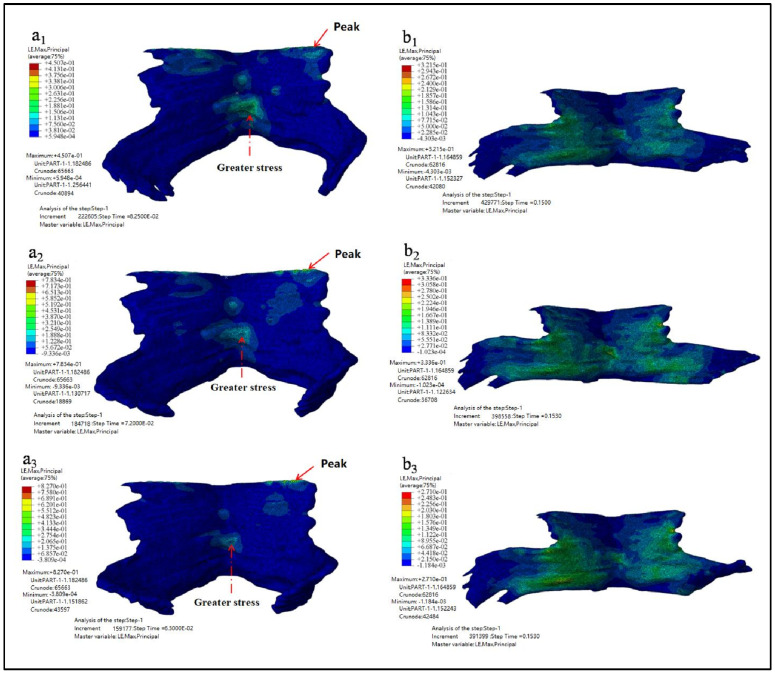
The maximum principal strain nephogram. (**a1**–**a3** showed the maximum principal strain nephogram of Models D80, D90, and D100 while **b1**–**b3** showed the principal strain nephogram of Models D80, D90, and D100 at simulation end time of 0.15 s).

Figure 8 showed the maximum principal strain nephogram of PFM and the principal strain nephogram at the end simulation time of 0.15 s. Figure 8(a1–a3) showed the maximum principal strain nephogram of Models D80, D90, and D100. It showed that the stress peak appears at the point of the straight-line arrow, while the stress value appears at the point of the dotted line arrow. Figure 8(b1–b3) showed the principal strain nephogram of Models D80, D90, and D100 at simulation end time of 0.15 s.

The maximum principal strain, the occurrence time of the maximum principal strain, and the principal strain value at the end of simulation are shown in Table 5.

As can be seen from the results listed in Table 5, the larger the biparietal diameter of the fetus is, the larger the maximum principal strain of PFM is. The peak value appears in the pubic coccygeus muscles, namely the place where PFM is attached to the skeleton. Secondly, the middle and lower part of the iliac tail seam also has a large strain. From the strain diagram of *t* = 0.15 s, it can be observed that there exists a large strain between the levator ani muscle and the pectineal coccygeus muscle.

**Figure 9:**
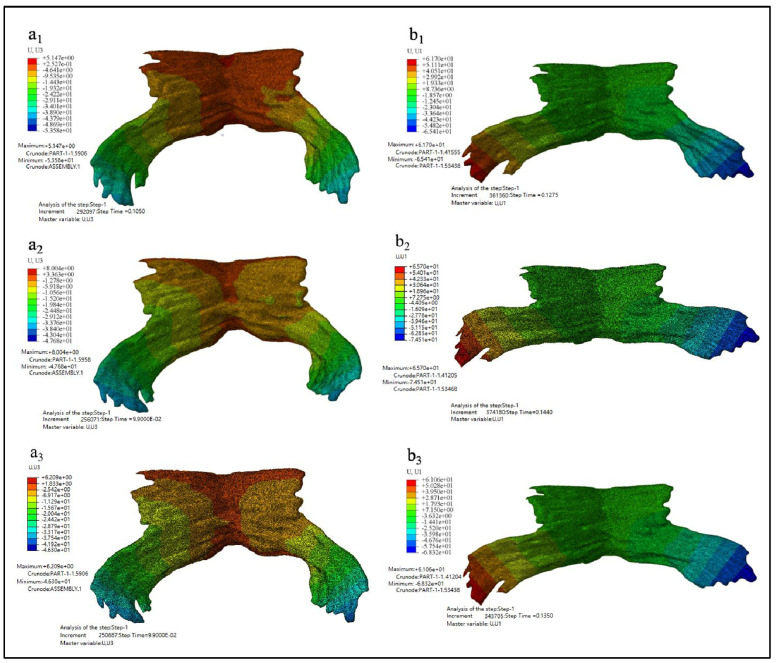
The displacement nephogram. (**a1**–**a3** showed the displacement nephogram in the Z direction, *u_Z_*, of Model D80, D90 and D100 while **b1**–**b3** showed the displacement nephogram in the X direction, *u_X_*, of Models D80, D90 and D100).

Figure 9 showed the displacement nephogram of PFM in Z direction and X direction. Figure 9(a1–a3) showed the displacement nephogram in the Z direction, *u_Z_*, of Model D80, D90 and D100. It reflects the stretching of the puboccygeus muscle. Figure 9(b1–b3) showed the displacement nephogram in the X direction, *u_X_*, of Models D80, D90 and D100. It reflects the stretching of the levator ani muscle.

The specific maximum displacement value and the occurrence time of the maximum displacement are shown in Table 6.

In Table 6, the maximum displacement in the X direction is the difference between the maximum and minimum displacement in the X direction. The extension ratio in the Z direction is equal to the ratio of the maximum displacement in the Z direction to the height of PFM, and the extension ratio in the X direction is equal to the ratio of the maximum displacement in the X direction to the width of PFM. It is known that PFM of this model is 40 mm height and 70 mm length.

The observed data show that the maximum displacement in the Z direction occurs at two-thirds of the total calculation time of the second stage of labor, and PFM is stretched in the vertical direction from 128.90% to 131.27%. This is mainly the ratio of the pectineal coccygeus muscle stretching, which indicates that during this period of time the region is most likely to develop muscle tears and organ prolapse. And the maximum displacement in the X direction occurs in the four fifths of the total calculation time of the second stage of labor, and PFM is stretched in the horizontal direction from 181.59% to 200.30% (which is mainly the stretching proportion of the levator ani muscle) and the distal displacement of the levator ani muscle is the largest. The results of comprehensive displacement nephogram showed that almost all the elements of PFM deform greatly during the whole second stage of labor.

## 4. Discussion

Results of this study showed that the levator ani and pubococcygeus muscle were the most stretched during vaginal delivery, which was partly consistent with Hoyte et al. study. They found that the maximum tension occurred in the anterior and lower part of the levator ani and also in the posterior and medial side of the pubococcygeus muscle during simulated delivery [24]. Jing et al. [17] found that when the fetal head moved along the Carus curve to about three-quarters of the total distance, the stress peak of levator ani was found, and at this time, the suboccipital orbital plane of the fetal skull and the diameter of twin occipital bone were passing through the levator ani hiatus. Secondly, the pectineus coccygeus is subjected to maximum tension and stress throughout labor. In the model of Parente et al. [27], when the vertical displacement of the fetal head reached about 60 mm, the maximum deformation of 66% was generated and the maximum tensile value was obtained. The maximum strain occurs in the region corresponding to the length of the levator ani and the pectineal coccygeus muscles.

In the results of this study, the peak stress and strain of PFM appeared at half of the simulation time, and the peak stress and strain appeared at the attachment point of pubic coccygeus muscle and skeleton, which is the same as Jing’s conclusion [17]. And in addition, during the simulation, the pubococcygeus muscle was stretched about 1.2 times in the Z direction, and the levator ani muscle was stretched more than two times in the X direction. Furthermore, the middle region of levator ani muscle and pubococcygeus muscle also has a large stress and strain, which is similar to the conclusion of Parente et al. [27]. The study of D”Aulignac et al. [26] has confirmed that the attachment point of pubococcygeus muscle and skeleton was the area where most postpartum lesions occur. It can be seen that the research results of this paper have a certain reference.

To sum up, during delivery, the whole PFM undergo huge deformation, among which the levator ani muscle and the pubococcygeus muscle are greatly stretched, and the attachment point between the pubococcygeus muscle and the skeleton is the place with the highest postpartum lesion probability.

The pelvic floor we studied here is a complex myofascial system, and thus several limitations involved must be clarified in order to properly interpret our findings. Firstly, although it is not true in reality, a relative simple pelvic floor muscle model was built in this study focused on investigating the characteristics of pelvic floor muscle motion during simulated labor with different fetal biparietal diameter sizes. The static force was applied as we assumed that the stretch was uniform and constant during the simulation. However, it may not affect our results as it has no influence on the inherent geometric difference between the size of fetal biparietal diameter and the prelabor urogenital hiatus [28,29]. Nevertheless, further research using the directional pelvic floor force is warranted for verification. Secondly, this is a pilot study in terms of PFM, and thus many underlying injury mechanisms, and even the detailed description of postpartum lesions (e.g., tears or avulsions and its functional repercussions) were not explored and involved. Last but the most important, due to technical restriction, it is always difficult or even impossible to verify the simulation results of the inner body parts through in vitro experimental tests [30,31]. Thus, the simulation results of this study were compared and verified with previous relevant studies for the overall trend and magnitude [17,18,26,27]. However, it may not be possible to compare the specific value due to different simulation settings and other potential factors. Future studies should devote to explore some parameters that can be tested through in vitro experiment, which could further help to verify the simulation results.

## 5. Conclusions

In conclusion, the present study showed that the peak stress and strain of PFM appeared at one-half of the delivery period, and at the attachment point of the pubococcygeus to the skeleton. During the simulation process of the second stage of labor, the pubococcygeus was stretched about 1.2 times and the levator ani muscle was stretched to more than two times. There existed also greater stress and strain in the middle area of levator ani muscle and pubococcygeus, contributing to postpartum lesions during delivery. In addition, the attachment point between the pubococcygeus and the skeleton were assumed the position with the highest probability of postpartum lesions. Our findings may provide simulation reference support for the prevention of pelvic floor lesions and treatment.

## Figures and Tables

**Table 1:** Model dimensions.

Pelvic Floor Muscle			Fetal Biparietal Diameter		
Length (mm)	Width (mm)	Height (mm)	Diameter D1 (mm)	Diameter D2 (mm)	Diameter D3 (mm)
70	60	40	80	90	100

**Table 2:** Material parameter list.

Part Name	Young Modulus (MPa)	Poisson Ratio	Density (ton/mm^3^)
Pelvic floor muscle	0.2	0.4	1.12 × 10^−9^
Fetal head	140,000	0.3	7.86 × 10^−9^

**Table 3:** Equivalent stress data sheet.

Fetal Biparietal Diameter D (mm)	Maximum Equivalent Stress (MPa)	Occurrence Time of the Maximum Equivalent Stress (s)	Equivalent Stress Value (*t* = 0.15 s) (MPa)
80	0.1494	0.0750	0.0782
90	0.1982	0.0900	0.0804
100	0.2218	0.1080	0.2509

**Table 4:** Principal stress data sheet.

Fetal Biparietal Diameter D (mm)	Maximum Principal Stress (MPa)	Occurrence Time of the Maximum Principal Stress (s)	Principal Stress Value (*t* = 0.15 s) (MPa)
80	0.1172	0.0825	0.0375
90	0.1941	0.0810	0.0575
100	0.2136	0.0540	0.1243

**Table 5:** Principal strain data sheet.

Fetal Biparietal Diameter D (mm)	Maximum Principal Strain	Occurrence Time of the Maximum Principal Strain (s)	Principal Strain Value (*t* = 0.15 s)
80	0.4507	0.0825	0.3215
90	0.7834	0.0720	0.3336
100	0.8270	0.0630	0.2710

**Table 6:** Maximum displacement.

Fetal Biparietal Diameter D (mm)	Maximum *u_Z_* (mm)	Extension Ratio in Z Direction	Occurrence Time of the Maximum *u_Z_* (s)	Maximum *u_X_* (mm)	Occurrence Time of the Maximum *u_X_* (s)	Extension Ratio in X Direction
80	50.3580	128.90%	0.1050	127.1100	0.1275	181.59%
90	50.6840	126.71%	0.0990	140.2100	0.1440	200.30%
100	52.5090	131.27%	0.0990	129.3800	0.1350	184.83%

## Data Availability

The data presented in this study are available on request from the corresponding author.
